# ASO Author Reflections: Can Utilization of Cancer Registry Data Contribute to Solving the Lack of Evidence for Older Pancreatic Cancer Patients?

**DOI:** 10.1245/s10434-020-08611-6

**Published:** 2020-05-27

**Authors:** J. V. Groen, C. J. H. van de Velde, E. Bastiaannet, J. S. D. Mieog

**Affiliations:** 1 Department of Surgery, Leiden University Medical Center, Leiden, The Netherlands; 2 Department of Medical Oncology, Leiden University Medical Center, Leiden, The Netherlands

## Past

Pancreatic cancer has a poor prognosis with a 5-year survival of approximately 7%.^1^ Only patients with stage I–II (localized disease) have a chance of long-term survival after resection. Recently, some advances were made in patients with localized disease who were treated with neoadjuvant chemoradiation therapy^2^ or adjuvant FOLFIRINOX^3^. Unfortunately, the median age of patients included in these randomized controlled trials (63–67) are not representative for the general pancreatic cancer population.^4^ Older patients are often not included in clinical trials, leading to a knowledge gap in treating older patients. The international European Registration of Cancer Care (EURECCA) project is a research committee supported by the European Society of Surgical Oncology. The aim of EURECCA is to utilize cancer registry data to compare and improve treatment strategies.^5^

## Present

In this international EURECCA study^6^, treatment strategies and survival outcomes of patients 70 years and older with stage I–II pancreatic cancer were compared in the Belgian, Dutch and Norwegian national cancer registries. Large differences were observed in the use of surgery and (neo)adjuvant and palliative chemotherapy. Only 23% of patients received the current standard-of-care (tumor resection preceded or followed by chemotherapy). Even stratified for treatment strategy, overall survival differed significantly between the cancer registries. Although this study provides no insight into quality of life, it appears that adequately selected older patients and more aggressive treatment can result in better overall survival.

## Future

Although the quantity and quality of randomized clinical trials is increasing^7^, we still expect that elderly patients will often be excluded. Therefore, the utilization of cancer registry data offers a solution in research of elderly patients. Another advantage over randomized clinical trials data, is that cancer registry data is readily available and population-based, thereby minimizing selection bias. EURECCA also aims to create awareness of the large variation in treatment strategies between cancer registries and generate new hypotheses for future research.^5^ Future studies are needed to identify selection criteria for local and systemic treatment, so that clinicians can offer tailored treatment to older patients with pancreatic cancer.

## References

[1] Lepage C, Capocaccia R, Hackl M (2015). Survival in patients with primary liver cancer, gallbladder and extrahepatic biliary tract cancer and pancreatic cancer in Europe 1999–2007: results of EUROCARE-5. Eur J Cancer.

[2] Versteijne E, Suker M, Groothuis K (2020). Preoperative chemoradiotherapy versus immediate surgery for resectable and borderline resectable pancreatic cancer: results of the Dutch randomized phase III PREOPANC Trial. J Clin Oncol.

[3] Conroy T, Hammel P, Hebbar M (2018). FOLFIRINOX or gemcitabine as adjuvant therapy for pancreatic cancer. N Engl J Med.

[4] Huang L, Jansen L, Balavarca Y (2018). Nonsurgical therapies for resected and unresected pancreatic cancer in Europe and USA in 2003–2014: a large international population-based study. Int J Cancer.

[5] Evrard S, van de Velde C, Noordhoek I (2019). European society of surgical oncology’s strategy for clinical research: paving the way for a culture of research in cancer surgery. Eur J Surg Oncol.

[6] Groen JV, Douwes TA, van Eycken E (2020). Treatment and survival of elderly patients with stage I–II pancreatic cancer: a report of the EURECCA pancreas consortium. Ann Surg Oncol.

[7] Huttner FJ, Capdeville L, Pianka F (2019). Systematic review of the quantity and quality of randomized clinical trials in pancreatic surgery. Br J Surg.

